# Orthologous nuclear markers and new transcriptomes that broadly cover the phylogenetic diversity of Acanthaceae

**DOI:** 10.1002/aps3.11290

**Published:** 2019-09-25

**Authors:** Erica B. Morais, Jürg Schönenberger, Elena Conti, Alexandre Antonelli, Péter Szövényi

**Affiliations:** ^1^ Department of Systematic and Evolutionary Botany University of Zurich 8008 Zurich Switzerland; ^2^ Department of Botany and Biodiversity Research University of Vienna Rennweg 14 A‐1030 Vienna Austria; ^3^ Gothenburg Global Biodiversity Centre Box 461 SE 40530 Göteborg Sweden; ^4^ Royal Botanic Gardens, Kew Richmond Surrey TW9 3AE United Kingdom

**Keywords:** Acanthaceae, Lamiales, nuclear markers, phylogeny, transcriptomes

## Abstract

**Premise:**

Information on orthologous groups of genes, their sequence variability, and annotation is required for project design in phylogenetic reconstruction. This resource is unavailable for the flowering plant family Acanthaceae (>4000 species).

**Methods:**

We compared transcriptome sequences spanning the extant diversity of Acanthaceae in order to provide a set of orthologous low‐copy nuclear genes and assess their utility for reconstructing phylogenetic relationships within this group of plants.

**Results:**

We present new transcriptome assemblies for eight species representing all major clades of Acanthaceae. The assemblies of five of these species are entirely based on new sequence data. Of these five species, three are from subfamilies for which no genomic resources were previously available (Nelsonioideae and Thunbergioideae). These five new transcriptomes are more complete than all others from public databases. Furthermore, we provide alignments with sequence information, annotation, and statistics for potential phylogenetic utility of 1619 orthologous low‐copy nuclear markers.

**Discussion:**

Our method of inferring assemblies from multiple pooled tissue samples delivers more complete transcriptomes than any available ones from Acanthaceae. We make available to the community new resources (e.g., sequence information, variability, and annotation of orthologous low‐copy nuclear genes) that will help phylogenetic reconstruction in Acanthaceae.

Acanthaceae belongs to the order Lamiales (>23,000 species [Chase et al., 2016]). It is among the 15 most species‐rich families of flowering plants (>4000 species) and is an ecologically and economically important clade, especially in the tropics (Tripp and McDade, 2014). Despite earlier efforts (e.g., Borg et al., 2008; McDade et al., 2008; Tripp and McDade, 2014), phylogenetic relationships within Acanthaceae remain contentious. In particular, the relationships among major evolutionary lineages within the family (e.g., the four subfamilies) as well as the generic limits within the largest subfamily (Acanthoideae) remain unresolved (e.g., McDade et al., 2018). Furthermore, the phylogenetic relationships in several smaller clades are currently not well understood (e.g., in *Avicennia* L. [Glasenapp et al., 2019], *Barleria* L. [Darbyshire et al., 2019], *Dyschoriste* Nees [Chumchim et al., 2015], Thunbergioideae T. Anderson [Borg et al., 2008], *Ruellia* L. [Tripp et al., 2018]). In addition to this obstacle, the rooting of the Acanthaceae phylogeny is uncertain, as interfamilial relationships in Lamiales remain elusive (Schäferhoff et al., 2010; Refulio‐Rodriguez and Olmstead, 2014; Stull et al., 2015; Wikström et al., 2015; Chase et al., 2016; Sarzi et al., 2019; Xu et al., 2019). These technical barriers prevent targeted investigations of evolutionary questions within this family.

Plant phylogenies are generally difficult to resolve. This may be due to several reasons, such as low number of substitutions observed in orthologous molecular markers (Li et al., 2019) and/or complex evolutionary histories (e.g., hybridization, polyploidization, complex gene history) not properly dealt with by the algorithms available for phylogenetic inference (e.g., most genes do not fit to any substitution model currently available; Sebastian Höhna, Ludwig‐Maximilians Universität, Munich, unpublished data). In addition, gene trees do not necessarily reflect species trees, which further complicates the inference of well‐resolved species phylogenies (e.g., Pease et al., 2016). Therefore, to build robust phylogenetic hypotheses, it is essential to compare the phylogenetic inference of several different genes. However, the lack of genomic resources (such as genomic and transcriptomic sequences) in Acanthaceae prevents sequencing of specific target loci. For instance, several molecular markers widely used for plant phylogenetics cannot be amplified in Acanthaceae species (A. J. Borg and J. Schönenberger, unpublished data). The methods currently accessible for sequencing molecular markers without prior sequence information of orthologous loci are either expensive (e.g., whole genome sequencing) or inappropriate (e.g., restriction site–associated DNA, which has low confidence for homology assessment [i.e., potential paralogy, high levels of missing data, and low reproducibility]) for phylogenetic inference at deeper phylogenetic levels or in older clades. The establishment of low‐copy nuclear genes (LCNG) suitable for phylogenetic analysis would help to further clarify the evolutionary history of Acanthaceae and of the Lamiales.

Compared to other currently used strategies of genome reduction prior to sequencing in plant systematics, high‐throughput targeted capture (Gnirke et al., 2009) offers several advantages (recently reviewed by Johnson et al., 2019) and has been widely applied in plant systematics and evolution. Transcriptome sequences have been successfully used to develop probe sets for targeting nuclear markers in several plant groups (Chamala et al., 2015; Landis et al., 2016; Crowl et al., 2017; García et al., 2017; Villaverde et al., 2018; Johnson et al., 2019; Vargas et al., 2019). The hybridization between RNA probes and DNA sequences is directly linked to their similarity. Hybridization leads to efficient target enrichment if sequence similarity between RNA probes and DNA sequences shows at least 85% similarity (Orin McCormick, RAPiD Genomics, unpublished data). Therefore, we decided to obtain sequence information for designing specific probes for Acanthaceae.

Currently, there is only a single draft genome published for Acanthaceae (*Ruellia speciosa* Mart. ex Nees, subfamily Acanthoideae; Zhuang and Tripp, 2017), but no genomic resources are available for the subfamilies Thunbergioideae and Nelsonioideae, which comprise some 180 and 172 species, respectively. To maximize the potential for successful hybridization to probe sequences from a set of phylogenetically diverse species such as the Acanthaceae (approximately 80 million years old; Tripp and McDade, 2014), it is critical to include a phylogenetically broad set of taxa when designing probes. In line with this, we generated new transcriptomic data for five species, representing all major clades of Acanthaceae (Fig. 1). Next, we compared our own sequences with transcriptomic data available for Acanthaceae in public data repositories (NCBI Resource Coordinators, 2016). We provide information on the utility for phylogenetic inference of orthologous loci within this plant group. This study provides a much‐needed set of nuclear markers that will facilitate phylogenetic reconstruction within the family Acanthaceae, as well as in Lamiales.

**Figure 1:**
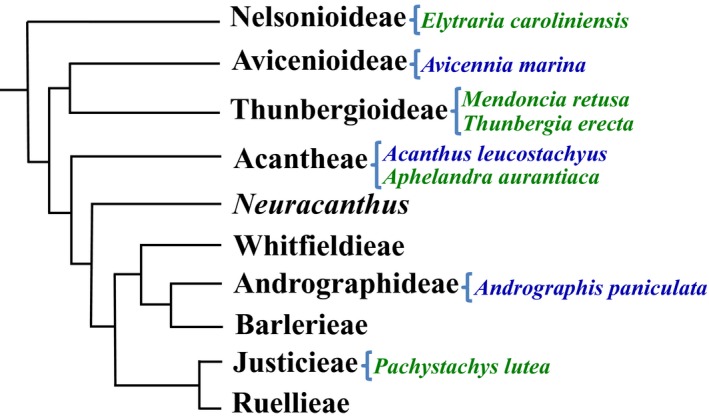
Phylogenetic relationships among major clades of Acanthaceae according to Tripp and McDade (2014). Names of species sampled are given on the right side. Taxa for which we provide new data are in green, and taxa for which we used GenBank data are in blue.

## METHODS

### Sampling

In order to sample all the major clades (the three first splits in the phylogeny of crown group Acanthaceae, according to Tripp and MacDade, 2014) within Acanthaceae (Fig. 1), we carried out RNA sequencing of five species and added data from three additional species from the National Center for Biotechnology Information Short Read Archive (NCBI SRA). We provide new transcriptomic data for two subfamilies lacking genomic resources: (1) Nelsonioideae (*Elytraria caroliniensis* (Walter ex J. F. Gmel.) Pers. and (2) Thunbergioideae (*Mendoncia retusa* Turrill and *Thunbergia erecta* (Benth.) T. Anderson). In addition, we sequenced two species that represent major lineages within the subfamily Acanthoideae, *Pachystachys lutea* Nees and *Aphelandra aurantiaca* (Scheidw.) Lindl. (voucher information given in Appendix 1). In order to further increase our sampling (Fig. 1) and to obtain more information on sequence variability, we also retrieved RNA sequencing data for the species *Acanthus leucostachyus* Wall. ex Nees (representing Acantheae), *Andrographis paniculata* (Burm. f.) Nees (representing Andrographideae), and *Avicennia marina* (Forssk.) Vierh. (representing Avicennioideae) from the NCBI SRA (Appendix 1). A recent phylogenomic study comprising Acanthaceae sensu stricto presents a different topology from the one presented by Tripp and McDade (2014) (Amanda Fisher, California State University, unpublished data). However, our sampling still comprises all major clades in the family according to this new topology.

### Sample preparation and sequencing

All tissues for RNA sequencing were freshly collected in botanical gardens (see Appendix 1 for voucher information). Vegetative and reproductive fresh juvenile tissues of each sample were flash‐frozen in liquid nitrogen or preserved in RNA*later* (Ambion, Waltham, Massachusetts, USA). We attempted to sample as much different young tissues/organs as possible in order to maximize the number of unique genes sequenced. Total RNA was extracted either using the NucleoSpin Plant RNA kit (Macherey‐Nagel, Düren, Germany) or the RNeasy Plant Mini Kit (QIAGEN, Hilden, Germany), following the manufacturer's recommendations (Appendix 1).

Illumina TruSeq Stranded mRNA (Illumina, San Diego, California, USA) libraries were prepared (with poly‐A RNA enrichment) and sequenced on an Illumina HiSeq2500 sequencer (HiSeq Control Software 2.2.58/RTA 1.18.64) with a 2 × 126‐bp setup using HiSeq SBS Kit v4 chemistry (run date 29 January 2016). We pooled all five samples on one lane (output of 249.31 million reads) of one flow cell to generate RNA‐Seq reads for each species.

### Data cleaning, transcriptome assembly, and annotation

Data quality was visually assessed with FastQC version 0.11.4 (Andrews, 2010) before and after data filtering and trimming. Adapter sequences (the first 13 base pairs), low‐quality reads (Phred score < 33), and reads shorter than 50 bp were removed with Trimmomatic‐0.35 (Bolger et al., 2014). The sequences were assembled into putative transcripts using Trinity version 2.1.1 (Haas et al., 2013). General statistics for quality assessment of transcriptome assemblies were obtained with the package GenomeTools (Gremme et al., 2013). We used the TRAPID pipeline (Van Bel et al., 2013) based on the PLAZA 2.5 database to get protein translations and to assess the number of fully or quasi–fully sequenced transcripts. We used BUSCO version 3.0.2 (Simão et al., 2015) with the embryophyte single‐copy ortholog set to assess the completeness of the transcriptome assemblies. All commands used for this study are available at Figshare (https://figshare.com/s/7c91497e3fb1cd0ceed7).

### Orthology assessment

To minimize the possibility of obtaining paralogous loci, we aimed at finding orthologous LCNG most appropriate for phylogenomic analyses. These genes are generally highly conserved and are, therefore, not ideal to resolve shallow phylogenetic relationships. However, they often contain introns with greater levels of variability, making them useful for a broad range of phylogenetic analyses even at low taxonomic ranks. We used MarkerMiner (Chamala et al., 2015) to establish groups of orthologous genes by using transcriptome assemblies and their protein translations as input. This approach uses the same predefined set of genes as a reference and has been successfully applied to capture sequences in other phylogenetic studies in angiosperms (e.g., Nicholls et al., 2015; Landis et al., 2016; Crowl et al., 2017; García et al., 2017; Villaverde et al., 2018; Vargas et al., 2019).

There is currently no taxon closely related to Acanthaceae with a well‐annotated high‐quality genome available. We were unable to use (with exonerate; Slater and Birney, 2005) the *Ruellia speciosa* genome (Zhuang and Tripp, 2017) as a reference in our analysis due to its low contiguity leading to many fragmentary gene models. Therefore, we decided to use the genome of *Arabidopsis thaliana* (L.) Heynh. as a reference. For each orthogroup recovered by MarkerMiner, we calculated statistics to estimate its phylogenetic utility (e.g., alignment length, number of variable sites, number of parsimony informative sites, AT and GC content) using AMAS (Borowiec, 2016). The output from MarkerMiner gives well‐annotated alignments for each orthogroup, including the boundaries of exonic regions in the assembled transcripts (alignments are available at https://figshare.com/s/9903aacaaa3c34bc9ed9).

## RESULTS

### Transcriptome assembly

We compared transcriptome assemblies (available at https://figshare.com/s/aa884dbe565dd1f453b2) of eight species of Acanthaceae, which represent all major clades (Fig. 1) within this family. Five of these species had no transcriptomic data resources previously available in public databases. Of these five species, three are from subfamilies (Nelsonioideae and Thunbergioideae) for which no genomic resources were available at all. Our transcriptome sequencing resulted in a total of 41–58 million raw reads per sample (Table 1). The assembly of quality‐filtered and trimmed reads produced 85,504–286,084 contigs per species. The functional annotation from TRAPID identified 7616–86,113 fully or quasi–fully sequenced transcripts per species (Table 1). Transcriptomes were 83–47.4% complete according to BUSCO (Table 2) (Simão et al., 2015). These five new transcriptomes are more complete than all others from public databases (Table 2).

**Table 1:** Quantity and quality information of assembled transcriptomes.

Species	Total no. of raw reads	No. of contigs	Mean contig length (bp)	Median contig length (bp)	No. of fully or quasi full‐length transcripts	Longest contig (bp)	Shortest contig (bp)
*Acanthus leucostachyus*	46,888,754	146,742	878.84	470	49,781	12,269	201
*Andrographis paniculata*	197,537,498	111,881	658.69	426	34,559	6695	201
*Aphelandra aurantiaca*	47,250,000	127,697	907.29	552	49,509	19,919	201
*Avicennia marina*	40,000,000	85,504	602.93	395	26,671	6706	201
*Elytraria caroliniensis*	58,040,000	175,062	959.82	591	7616	15,753	201
*Mendoncia retusa*	49,650,000	260,725	777.2	457	37,428	16,484	201
*Pachystachys lutea*	41,080,000	115,380	974.38	584	45,408	14,585	201
*Thunbergia erecta*	53,290,000	286,084	769.81	450	86,113	16,502	201

**Table 2:** Assessment of transcriptome completeness based on 1440 universal single‐copy orthologs (BUSCO).^a^

Taxon	Complete	Complete and single‐copy	Complete and duplicated	Fragmented	Missing
Acanthus leucostachyus*	78.0%	44.7%	33.3%	6.0%	16.0%
Andrographis paniculata*	59.4%	42.7%	16.7%	16.2%	24.4%
*Aphelandra aurantiaca*	82.3%	44.7%	37.6%	5.6%	12.1%
Avicennia marina*	47.4%	36.8%	10.6%	20.6%	32.0%
*Elytraria caroliniensis*	84.3%	28.5%	55.8%	6.0%	9.7%
*Mendoncia retusa*	82.5%	35.1%	47.4%	6.5%	11.0%
*Pachystachys lutea*	83.5%	50.0%	33.5%	5.8%	10.7%
*Thunbergia erecta*	83.0%	34.2%	48.8%	6.6%	10.4%

a Species names with asterisks refer to data downloaded from the National Center for Biotechnology Information; species without an asterisk refer to transcriptomes generated in this study.

### Phylogenetic utility

We found 1619 putative orthologous LCNGs for Acanthaceae (alignments available at https://figshare.com/s/9903aacaaa3c34bc9ed9). Here we provide sequence information for bait design in Acanthaceae, offering flexibility of choice based on variability, presence or absence of species, intron size, and gene size. We make available the set of 1619 alignments from which baits are designed. On average, 3.68 species occurred in each of the orthogroups, which exhibited zero to 0.673 variable sites per position (0.448 on average, median value 0.411). We found 50 orthogroups that included transcripts for all species (eight). This number has increased when orthogroups were required to contain sequence data for at least seven, six, five, and four species (160, 369, 590, and 840, respectively). Parsimony informative sites per orthogroup ranged from zero to 2603 (362 on average, median value 225) (statistics for potential phylogenetic reconstruction available at https://figshare.com/s/ebb5b55c721debdaccb4).

## DISCUSSION

The exons of all LCNGs (except nine that do not have any variable site: AT1G21370, AT1G31500, AT1G79120, AT3G25530, AT4G01030, AT4G18975, AT4G28830, AT4G38370, and AT5G14140) were found have the potential to solve phylogenetic relationships at deeper nodes (see alignments available at https://figshare.com/s/9903aacaaa3c34bc9ed9). We observed that exons are more conserved among species of the same major clade of Acanthaceae (e.g., *Mendoncia* and *Thunbergia*, or *Acanthus* and *Aphelandra*). The variation within Thunbergioideae (*Mendoncia* and *Thunbergia*) is even lower. In order to resolve relationships among closely related species, targeting flanking regions of exons is an efficient approach to sequence the more variable introns (if the DNA is degraded and sequence reads are short, shorter introns are easier to capture and to sequence), which likely provide more phylogenetic information within the Acanthaceae. In this case, longer sequencing reads are desired to sequence introns captured by RNA baits, which are usually designed for exonic regions. Designing RNA primers for PCR is an alternative to hybrid capture. This method is efficient for amplifying long genes, including the introns (e.g., Valderrama et al., 2018).

Vargas et al. (2019) published a python script (GoldFinder) to sub‐select markers from the output of MarkerMiner (alignments available at https://figshare.com/s/9903aacaaa3c34bc9ed9) according to five relevant criteria for most users who work with molecular phylogenetics and evolution: (1) marker length, (2) percentage of short exons (relative to bait length), (3) number of user's sequences per marker, (4) similarity, and (5) bait number, length, and coverage. GoldFinder makes the sub‐selection task automatic and informed, so that it could be easily run using the data provided on Figshare (https://figshare.com/s/9903aacaaa3c34bc9ed9).

Johnson et al. (2019) developed a universal probe set for targeted sequencing of 353 nuclear genes from any angiosperm. However, the efficiency of hybridization of this probe set varied considerably across the different species tested and in some species/clades it was rather low (e.g., 5%, median for all samples was 24.8%). Johnson et al. (2019) selected sequences with up to 30% divergence to design their probes. Accordingly, the capture efficiency for these targets will most likely be improved by designing baits more specific for Acanthaceae, which have not been included in their study. The transcriptome sequences we make available here are a crucial resource for this purpose and can be used to further improve universal probe sets, such as the one by Johnson et al. (2019).

Our method of inferring assemblies from multiple pooled tissue samples delivers more complete transcriptomes than any previously available from Acanthaceae. In addition to being useful for phylogenetic analyses (the main goal of this study), the data generated here provide a potentially important basis for a wide array of other research projects, such as population genomic analyses, metabolic pathway investigations, gene prediction, crop improvement, and analyses of phenotypic diversity. Here, we provide a comparative analysis of representatives of Acanthaceae with the necessary tools for RNA bait design.

## AUTHOR CONTRIBUTIONS

E.B.M. designed the project, carried out data acquisition and analyses, and wrote the manuscript. J.S. helped to develop the study and supervised the design. P.S. supervised data analyses and writing. All authors have read and revised various versions of the manuscript.

## Data Availability

The following data are available on Figshare: transcriptome assemblies (https://figshare.com/s/aa884dbe565dd1f453b2), alignments for each low‐copy nuclear gene (input for baits design) (https://figshare.com/s/9903aacaaa3c34bc9ed9), statistics describing their potential for phylogenetic reconstruction (https://figshare.com/s/ebb5b55c721debdaccb4), and commands used to perform analyses (https://figshare.com/s/7c91497e3fb1cd0ceed7). Raw sequence data are available in the National Center for Biotechnology Information Sequence Read Archive (accession numbers are shown in Appendix 1).

## APPENDIX 1 Sample details for taxa used in this study.

**Table:**

Taxon	Tissue	Fixation method	Extraction method	Voucher^a^	SRA accession no.	Botanical garden
*Acanthus leucostachyus* Wall. ex Nees	L, R	—	—	—	SRR1793319 *	—
*Andrographis paniculata* (Burm. f.) Nees	L	—	—	—	SRR1292497 *	—
*Aphelandra aurantiaca* (Scheidw.) Lindl.	L, Fl	RNA*later*	QIAGEN	E.B. Morais 168, WU 0104498	SRR8782583	University of Vienna
*Avicennia marina* (Forssk.) Vierh.	L	—	—	—	SRR653719 *	—
*Elytraria caroliniensis* (Walter ex J. F. Gmel.) Pers.	L, Fl	Liquid nitrogen	QIAGEN	E.B. Morais 163, WU 0104492	SRR8756096	University of Vienna
*Mendoncia retusa* Turrill	S, L, Fl	Liquid nitrogen	Macherey‐Nagel	A.J. Borg 1, S or Z 000172480	SRR8749657	University of Stockholm
*Pachystachys lutea* Nees	S, L, Fl	RNA*later*	QIAGEN	E.B. Morais 164, WU 0104493	SRR8755478	University of Vienna
*Thunbergia erecta* (Benth.) T. Anderson	S, L, Fl	Liquid nitrogen	Macherey‐Nagel	E.B. Morais 173, Z 000172481	SRR8752193	University of Gothenburg

Note

— = not available; Fl = flower; L = leaf; R = root; S = stem.

a Herbaria abbreviations follow Index Herbariorum (see http://sweetgum.nybg.org/science/ih/).

* Data downloaded from the National Center for Biotechnology Information.
